# Supplementary material for: N2FXm, a method for joint nuclear and cytoplasmic volume measurements, unravels the osmo-mechanical regulation of nuclear volume in mammalian cells
Source: Nat Commun. 2024 Feb 7;15:1070. doi: 10.1038/s41467-024-45168-4 (PMC10850064; doi:10.1038/s41467-024-45168-4)
Supplement: Supplementary file 1 — Supplementary Information [file 41467_2024_45168_MOESM1_ESM.pdf]

Supplementary Materials for

N2FX: a method for joint nuclear and cytoplasmic volume measurements unravels the osmo-mechanical regulation of nuclear volume in mammalian cells.

Author list: Fabrizio A. Pennacchio[1,2], Alessandro Poli[1], Francesca Michela Pramotton[3], Stefania Lavore[1], Ilaria Rancati[1], Mario Cinquanta[1], Daan Vorselen[4], Elisabetta Prina[1], Orso Maria Romano[1], Aldo Ferrari[3], Matthieu Piel[5,6], Marco Cosentino Lagomarsino[1,7], Paolo Maiuri[1,8]*

Correspondence to: paolo.maiuri@unina.it

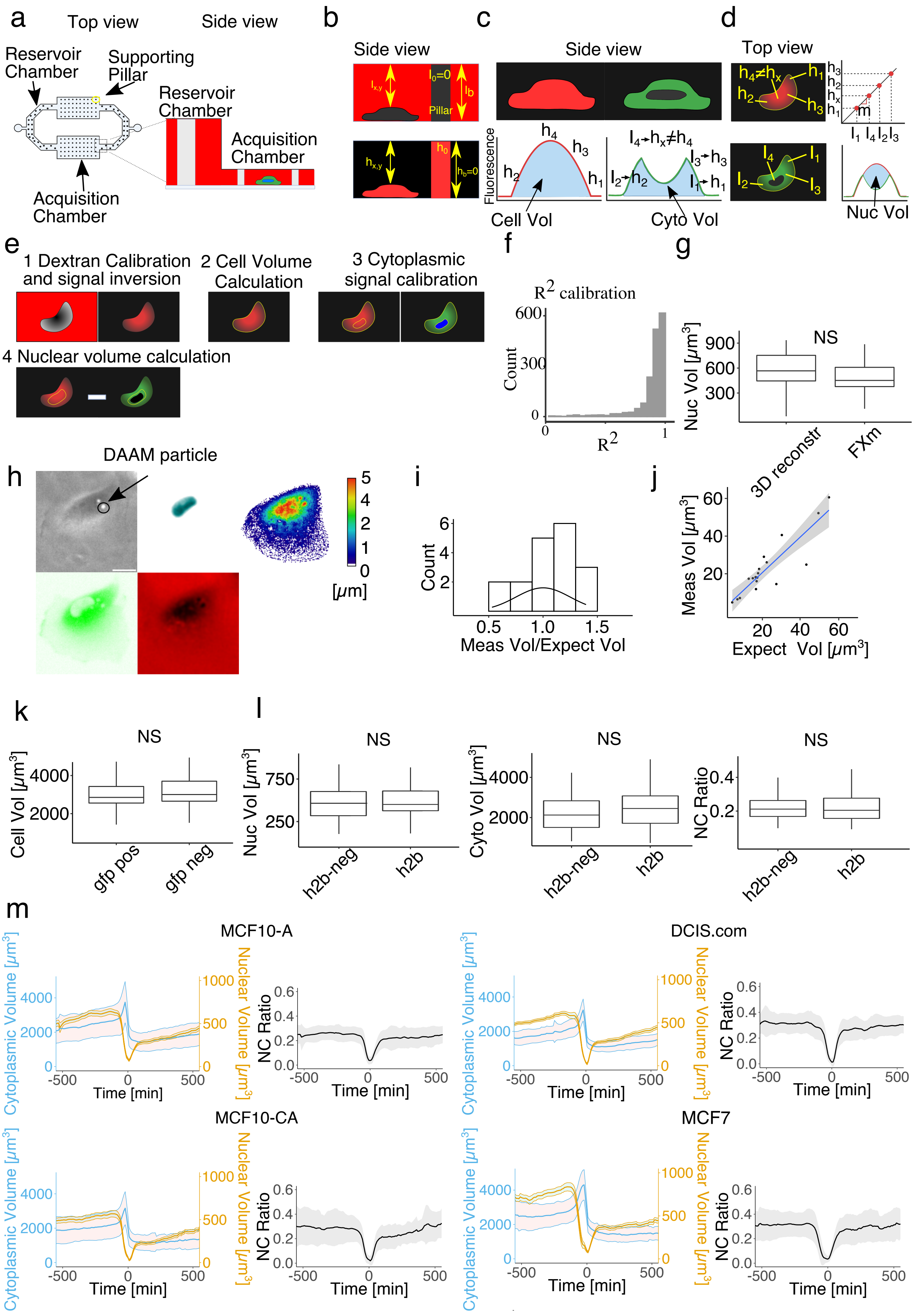
a
Top view
Side view
Reservoir Chamber
Supporting Pillar
Reservoir Chamber
Acquisition Chamber
Acquisition Chamber
b
Side view
Pillar
c
Side view
Fluorescence
Cell Vol
Cyto Vol
d
Top view
Nuc Vol
e
1 Dextran Calibration and signal inversion
2 Cell Volume Calculation
3 Cytoplasmic signal calibration
4 Nuclear volume calculation
f
R2 calibration
Count
g
NS
Nuc Vol [μm3]
3D reconstr
FXm
h
DAAM particle
[μm]
i
Count
Meas Vol/Expect Vol
j
Meas Vol [μm3]
Expect Vol [μm3]
k
NS
Cell Vol [μm3]
gfp pos
gfp neg
l
NS
Nuc Vol [μm3]
h2b-neg
h2b
NS
Cyto Vol [μm3]
h2b-neg
h2b
NS
NC Ratio
h2b-neg
h2b
m
MCF10-A
DCIS.com
MCF10-CA
MCF7
Cytoplasmic Volume [μm3]
Nuclear Volume [μm3]
NC Ratio
Time [min]

Fig. S1. Technique development and mean volume curves generation: (a) Sketch of the microfluidic device for FXm acquisition (top and side view). In the side view, the connection between the reservoir chamber and the acquisition chamber is showed. Schematic representation of: (b) the first calibration route for cell volume measurement, (c) the calibrated cell height profile (left) and the non-calibrated cytoplasmic height profile (right), (d) the second calibration route and nuclear volume calculation (bottom right). (e) Sketch of the image analysis workflow: (1) first calibration is performed, (2) by defining cell edges from the cytoplasmic staining, cell volume is calculated, (3) combining nuclear and cytoplasmic fluorescent signals, the cytoplasmic region between cell edges and the nuclear region is automatically defined and the second calibration is then performed, (4) nuclear volume is computed by subtraction considering both cell and cytoplasmic calibrated signals in a region equal or bigger than the nuclear region (user defined). (f) Cumulative distribution over several frames (>1500) and different cells (10) of R^2 relative to linear fits of pixels GFP-NES intensity in the cytoplasm in function of the corresponding pixel optical height computed with traditional FXm. (g) Nuclear volume distributions comparison considering volumes measured with the FXm (n=95) or with confocal 3D reconstruction (n=50). Welch Two Sample t-test gave p=0.30. (h) 10x image of a cell internalizing DAAM particles. Here, transmission, BFP, GFP, Texas red and the associated calibrated image (on the right) of a cell are showed. Scale bar 20 μm. (i) Distribution of measured on expected values ratio (n=18). Measured on expected value ratio distribution was compared with a normal distribution centered on 1 with a one sample t-test, giving a p value=0.39 and indicating that the error relative to our measurement was not systematic. (j) Scatter plot of DAAM particle "measured vs expected" volumes. Linear fit coefficients: slope=0.94, Pearson correlation coefficient=0.89, R^2=0.78. To assess if the introduction of exogenous fluorescent proteins (i.e. GFP-NES and H2B-BFP co-infection) altered cellular dimensions, we compared cell volume of differently stained cells. To evaluate eventual effects of the GFP-NES, we compared cell volumes of stained (n=86) and non-stained (n=42) populations. Both cell-types were analyzed in the same device and during the same acquisition. Cell volume was evaluated for all the tested cells 480 min before cytokinesis. Welch Two Sample t-test gave p=0.39 (k). For what concern nuclear staining, instead, we compared nuclear, cytoplasmic volumes and the relative nc ratio distributions of double stained cells (2NES-GFP and H2B-BFP, our control, n=95) with cells presenting only the cytoplasmic staining (H2B-neg, n=91). We did

not found significant differences (p=0.089 and p=0.32, p=0.11, respectively) (l). (m) Mean volume and mean NC ratio trajectories across division of MCF10-A (n=109), DCIS.com (n=78), MCF10-CA (n=82) and MCF7 (n=62) cells. Time frame= 10 min. Mean ± SD. Source data are provided as a Source Data file. “n” represents the number of cells examined over at least 3 independent experiments.

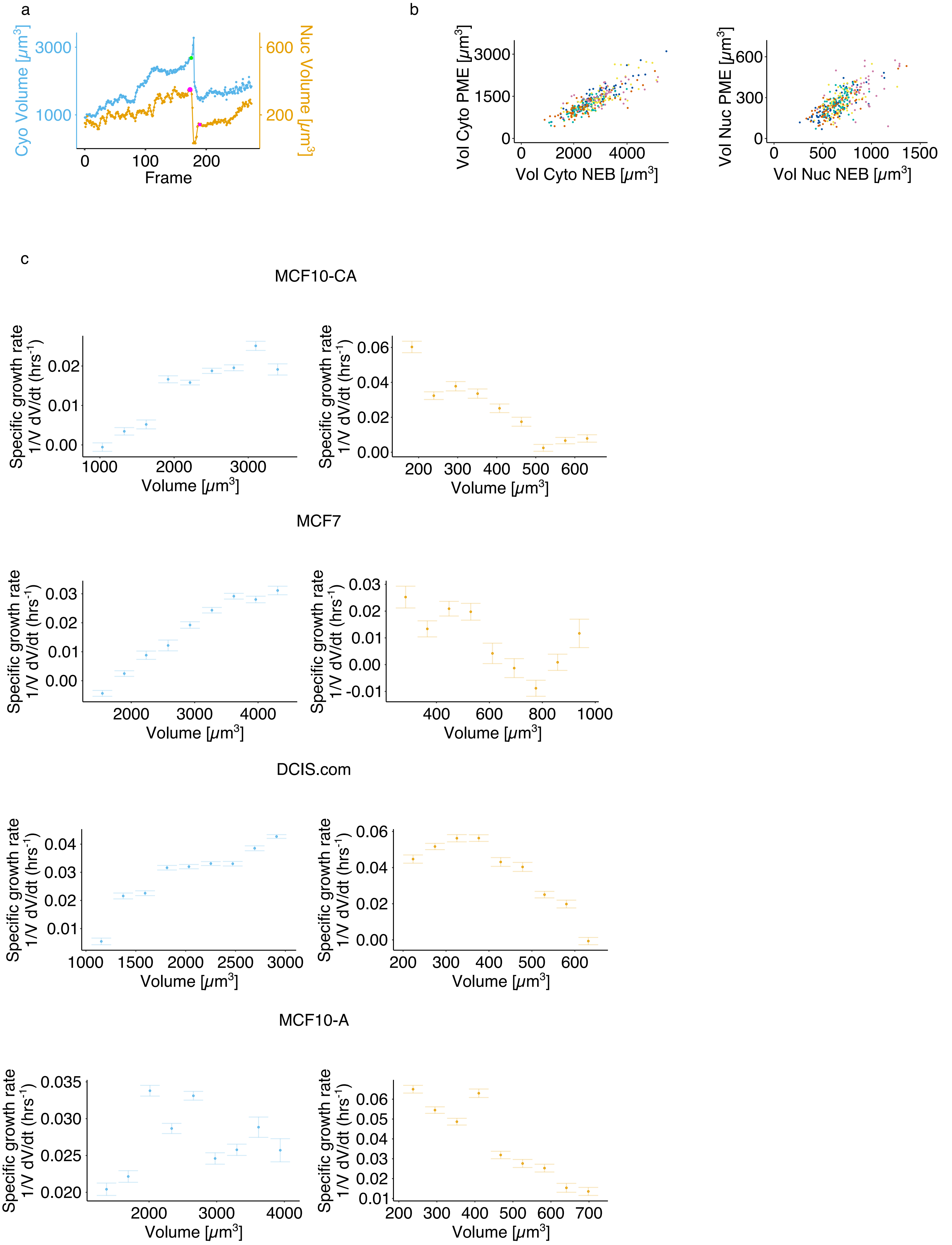
a
b
c
Cyo Volume [μm3]
Nuc Volume [μm3]
Frame
Vol Cyto PME [μm3]
Vol Cyto NEB [μm3]
Vol Nuc PME [μm3]
Vol Nuc NEB [μm3]
MCF10-CA
MCF7
DCIS.com
MCF10-A
Specific growth rate 1/V dV/dt (hrs-1)
Volume [μm3]

Fig. S2. Dynamic analysis and growth curves generation: (a) Point detection example on a single cell trajectory. Here, points in magenta on the nuclear volume curve represent NEB (before division) and PME (after division), respectively, while green point on the cytoplasmic curve represents the roundup onset. (b) Scatter plot linking the cytoplasmic (left) and nuclear (right) volumes at NEB and PME. (c) MCF10-CA, MCF7, DCIS.com and MCF10-A cytoplasm (left, blue) and nucleus (right, yellow) specific volume growth rate, defined as the binned average of $\frac{1}{V} \cdot \frac{dV}{dt}$ at fixed volume V, plotted as a function of volume (mean ±SE). In (b): RPE1 n=82, MCF10-A n=88, DCIS.com n=66, MCF10-CA n=66, MCF7 n=42. In (c): MCF10-A n=109, for DCIS.com n=78, for MCF10-CA n=82 and for MCF7 n=62. Mean ± SE. Source data are provided as a Source Data file. "n" represents the number of cells examined over at least 3 independent experiments.

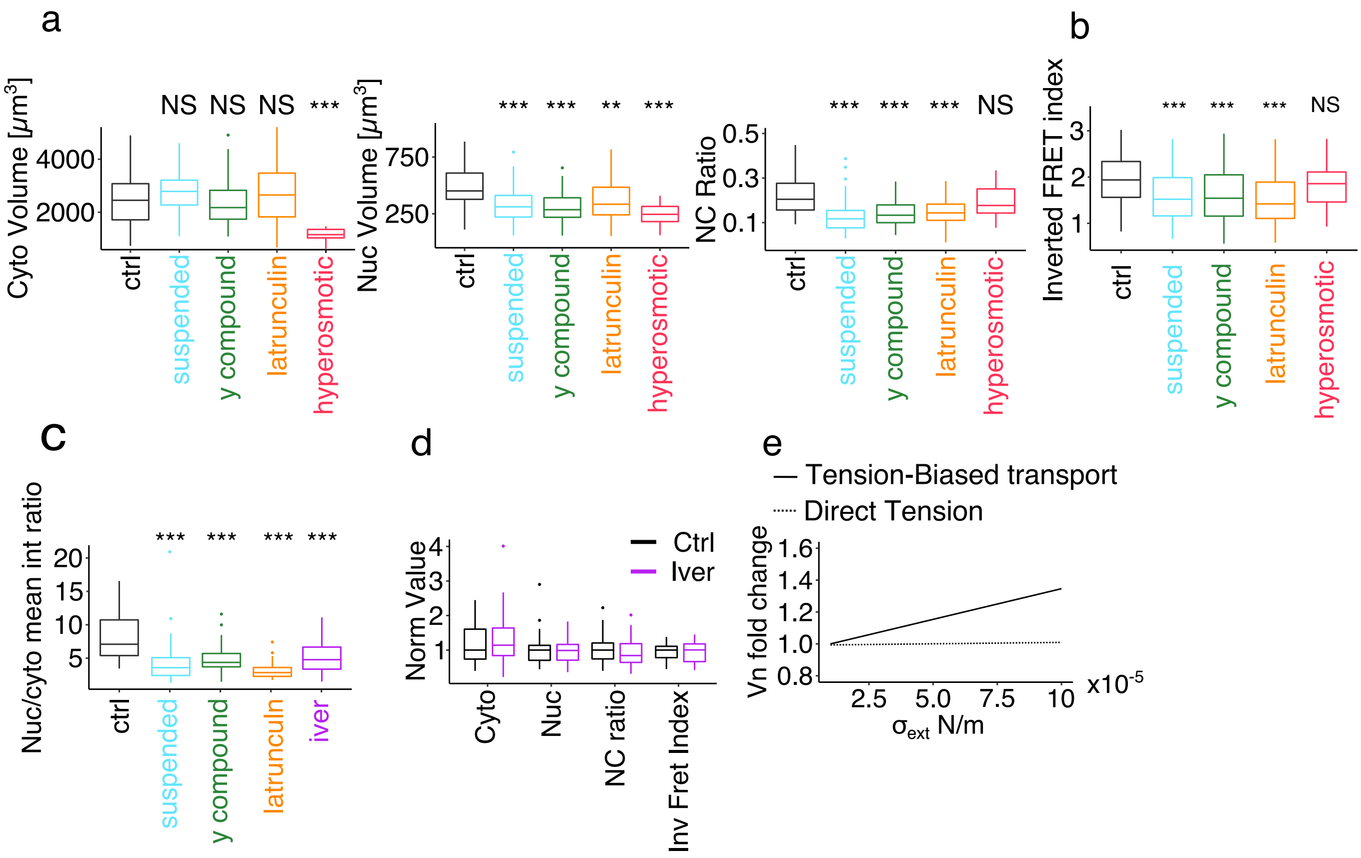
a
Cyto Volume [μm3]
Nuc Volume [μm3]
NC Ratio
NS NS NS ***
*** *** ** ***
*** *** *** NS
ctrl
suspended
y compound
latrunculin
hyperosmotic
b
Inverted FRET index
*** *** *** NS
c
Nuc/cyto mean int ratio
*** *** *** ***
iver
d
Norm Value
Ctrl
Iver
Cyto
Nuc
NC ratio
Inv Fret Index
e
Tension-Biased transport
Direct Tension
Vn fold change
σext N/m
x10-5

Fig. S3. Mechanical nuclear volume regulation: (a) Nuclear and cytoplasmic volumes distribution for untreated adherent cells (ctrl, n= 95), untreated detached cells (n= 77, p_{nuc}= 0.00014, p_{cyto}= 0.071, p_{ratio}= $2.35 \cdot e^{-7}$), Y27 treated cells (n= 64, p_{nuc}= $1.54 \cdot e^{-6}$, p_{cyto}= 0.89, p_{ratio}= $3.90 \cdot e^{-10}$), latranculin treated cells (n= 85, p_{nuc}= 0.0070, p_{cyto}= 0.16, p_{ratio}= $2.55 \cdot e^{-5}$ and for cells subjected to hyperosmotic shocks (n=24, p_{nuc}= $2.27 \cdot e^{-13}$, p_{cyto}= $2.41 \cdot e^{-9}$, p_{ratio}= 0.070). (b) Inverted FRET index (i.e. indirect measurement of nuclear envelope tension) distribution for untreated adherent cells (ctrl, n= 156), untreated suspended cells (n= 91, p=$9.66 \cdot e^{-7}$), Y27 treated cells (n= 119, p= $3.57 \cdot e^{-8}$), latranculin treated cells (n=129, p= $1.58 \cdot e^{-10}$) and for cells subjected to hyperosmotic shocks (n= 92, p= 0.051). Here, Y27 compound and latranculin were used to assess the effects related of stress fibers and contractility, respectively. (c) Distribution of the nucleus on cytoplasm ratio of the GFP mean intensity for untreated adherent cells (n=58), untreated detached cells (n=38, p=$2.07 \cdot e^{-11}$), Y27 treated cells (n=44, p=$4.52 \cdot e^{-08}$), latranculin treated cells (n=45,p=$2.2 \cdot e^{-16}$) and ivermectin treated cells (n=44, p=0.00016) (D) Normalized volumes (nuclear and cytoplasmic), nc ratios and Inverted FRET index distributions relative to untreated (black) and ivermectin (magenta) treated cells. Data were normalized for the median/mean of the considered variable (i.e. nuclear volume, cytoplasmic volume, nc ratio, inverted FRET index), which was calculated over the control dataset. Here, n_{ctrl} = 45, n_{iver} = 45. p_{nuc}= 0.19, p_{cyto}=0.83, p_{ratio}= 0.16 for volumes data. For Inverted FRET index distribution n_{ctrl}= 56, n_{iver}= 85, p=0.95. Statistical analysis were performed by means of the welch two sample t-test function in R. NS for p>0.05, * for p<0.05, **for p<0.01, *** for p<0.001. (e) Quantitative prediction of the developed theoretical model describing the effects on nuclear volume mediated by a transport biased mechanism (continuous line) or by a direct force (dashed line). The plot reports the predicted fold change in nuclear volume (y axis) for increasing applied extensile tension σ_{ext}. Parameter values: cytoplasmic volume V_C = 2000 μm^3, 20% fraction NLS/NES proteins in absence of applied tension, and a tension-transport bias of $\eta = 4 \cdot 10^3$ m/N (estimated from Andreu et al. 2021 (26), see SI appendix). This model neglects direct effect of extensile tension on nuclear size. The dashed line instead refers to the direct effects of applied extensile tension, which lead only to a mild increase of nuclear volume. The plot reports the predicted fold change in nuclear volume (y axis) at fixed cytoplasmic osmotic pressure for increasing applied extensile tension σ_{ext}. In the plot, nuclear volume increases only by 0.1%. Parameter values: α = 2/3 (fraction of osmotically active proteins that are in the cytoplasm), basal nuclear surface

tension $\sigma_0 = 10^{-6}$ N/m, cytoplasmic osmotic pressure $\Pi_C = 4$ kPa. See SI appendix for a detailed discussion of the models and the parameters. Source data are provided as a Source Data file. “n” represents the number of cells examined over at least 3 independent experiments.

SI appendix for Pennacchio et al. Osmo-mechanical equilibrium model for nuclear volume

This SI appendix describes the mathematical model used to complement our understanding of our experimental data.

Model ingredients

The key ingredient of the model is the assumption that osmotic pressure plays a primary role in setting nuclear and cytoplasmic volume (Mitchison, 2019). We use a bag in a bag model, similar to refs (Deviri and Safran, 2022; Lemière et al., 2022). Following these studies, we assume a spherical cell and nucleus, and that on the obervation time scales both the cell surface and the nuclear surface are at mechanical equilibrium (Salbreux et al., 2007; Venkova et al., 2022). Under these assumptions, a first equation describes the force balance between osmotic pressure and mechanical tension at cell membrane,

$$k_B T \left(\frac{N_C}{V_C^{(e)}} - c_{\text{out}}^{\text{macro}} + \frac{N_I}{V_C^{(e)}} - c_{\text{out}}^{\text{ions}} \right) = \frac{2\sigma_C}{R_C} \, , \tag{1}$$

In this equation, the right hand side ($\Delta\Pi$) is the osmotic pressure, while the left-hand side (ΔP) is the contribution of mechanical forces (in our case, surface tension and the cytoskeleton). In this equation $V_C^{(e)}$ is the accessible cellular volume contributing to osmosis, R_C is the radius of the cell, N_C is the number of osmotically active macromolecules in the cell (which might comprise macromolecules as well as small molecules (Rollin et al., 2023)), while N_I is the number of osmotically active ions in the cell, balancing respectively $c_{\text{out}}^{\text{macro}}$ and $c_{\text{out}}^{\text{ions}}$ the external concentrations of macromolecules and ions. Finally, σ_C is a mechanical surface tension of the cell. A variant of this equation can consider also the role of osmotically active small osmolytes.

A second equation following the same principles describes mechanical equilibrium between osmotic pressure and mechanical tension at the nuclear envelope. The nucleus has pores, hence we can safely neglect ions and small osmolytes, and assume that only macromolecules are osmotically active for this compartment (Deviri and Safran, 2022) (see also below)

$$k_B T \left(\frac{N_N}{V_N^{(e)}} - \frac{\alpha N_C}{V_C^{(e)}} \right) = \frac{2\sigma_N}{R_N} = \frac{2(\sigma_0 + \sigma_{\text{ext}})}{R_N} \, , \tag{2}$$

where the notation is similar as Eq. (1), and the suffix N stands for nucleus. We note that N_N is the number of osmotically active proteins in the nucleus (roughly, those that have an NLS), equally, we assume that αN_C is the number of osmotically active proteins in the cytoplasm (this is different from N_C, which also includes proteins that can freely shuttle). Finally we have decomposed the tension term into σ_0, a (constitutive) nucleus surface tension and an active contribution σ_{ext} from external forces from the cytoskeleton or other active elements, of which we want to test the quantitative effects. This tension of external origin can be related to the total magnitude of an external radial force required to generate the equivalent mechanical pressure difference. As we assume spherical symmetry, σ_{ext} is linked to the modulus of such an external constant radial external force by the relation $F_{\text{ext}} = -8\pi R_N \sigma_{\text{ext}}$ (where the minus sign indicates that a positive tension contributes to an inward force). Equation (2) can be seen as a simplified version of the model by Kim *et al.* (Kim et al., 2015), neglecting mechanics of nuclear shape deformations.

A recent study (Andreu et al., 2021) finds that nuclear import increases under force, while export is not biased. Motivated by this, and by our experimental observations, we also propose and examine a model variant incorporating the indirect effects of this force-biased transport on the equilibrium nuclear size. We model this effect in our simple framework as a bias on the nuclear concentration of proteins, based on the external tension, following

$$c_N = c_N^{(0)} e^{\eta \sigma_{\text{ext}}} \ , \tag{3}$$

where $c_N = N_N / V_N^{(e)}$ and η is a coefficient that quantifies the bias. In the above expression, we have used an exponential dependency under the assumption that the force-biased transport might be an activated process. However, we point out that to our scopes an assumption of linear response would be completely equivalent (see below). We estimate the value of η directly from experimental data from ref. (Andreu et al., 2021) below.

The main assumption of this model is that cell and nucleus are always in mechanical equilibrium, because the observation time is longer than the relaxation time scales. Additionally, we neglect tension terms for the cell ($\sigma_C \approx 0$), these terms should be small following Deviri and Safran, although they may lead to corrections (Deviri and Safran, 2022). For simplicity, we also always assume spherical symmetry, neglecting any mechanical deformation. These radical assumptions make the model treatable, and should not affect our conclusions, which are based on qualitative behavior or on order-of-magnitude quantitative differences. In standard conditions, external macromolecular osmotic (oncotic) pressure on the cell should be negligible. $c_{\text{out}}^{\text{macro}} \approx 0$, since the hyperosmotic shocks are applied with sucrose, this term becomes relevant in that case (the relevant quantity should be $c_{\text{out}}^{\text{macro}} + c_{\text{out}}^{\text{ions}} c_{\text{out}}$. Eq. (2) neglects ions (chromatin counterions) for the nucleus, a small contribution according to Deviri and Safran (Deviri and Safran, 2022), although the counterions might affect cell volume depending on chromaitn condesation (Rollin et al., 2023), an effect that we neglect here. Finally, we neglect non-osmotically accessible volume for the nucleus (not measured in our data), $V_N^{(e)} = V_N$ and we assume that for the cell the osmotically accessible volume is the cytoplasmic volume V_C. A variant considering inaccessible volumes as parameters is considered by Deviri and Safran, who show that their predictions are robust. In any case these corrections do not affect qualitative behavior or vary the quantitative behavior by orders of magnitude.

Under these assumptions, the model behavior is simple. External osmotic pressures (and mechano-osmotic coupling from pumps and channels) set a cell volume, and we can envisage a situation where the cell is (essentially) causally uncoupled from the nucleus, and solve Eq. (1) for the cytoplasmic osmotic pressure or equivalently cytoplasmic volume. Then these variables can be used as input variables for the nucleus, solving Eq. (2). While highly simplified - this picture is sufficient to explore different scenarios for our data, as we detail below.

Estimates of relevant parameters

All the key parameters and/or parameter ranges were fixed from literture values. Osmotic pressures Π_c and Π_n are order 10000 Pa (N/m^2) (Deviri and Safran, 2022). In order confirm this estimate, we can use the fact that volumes are in the range of 100-1000 μm^3, while protein concentrations are known to be in the range of $2-4 \cdot 10^6$ molecules / μm^3, which means that in a compartment of $\sim 300\mu$m^3 such as the nucleus there are about $3 \cdot 10^8$ proteins, and in a compartment of $\sim 2000\mu$m^3 such as the cytoplasm there are about $2 \cdot 10^9$ (to which we have to sum the contribution of the small molecules). Below there are some arguments on how many of them should be osmotically active (Milo, 2013). Assuming that a certain number of molecules (probably order $10^8 - 10^9$ given the above considerations) are osmotically active in a compartment, we can obtain osmotic pressures ($\Pi = k_B T N/V$). For example for a nucleus of $\sim 300\mu$m^3 with $3 \cdot 10^8$ proteins we get a proessure of 4 kPa. Once again, the estimates are

not counting the role of ions and small osmolites in the cytoplasm, but they should be reliable for the nucleus (and a lower bound for the cytoplasm).

Previous studies suggest that about 80% of the proteins are estimated to be localized (either in the nucleus or in the cytoplasm) (Deviri and Safran, 2022). Hence, we estimate that $(\alpha N_C + N_N)/(N_C + N_N) = 0.8$, which gives $\alpha \approx 2/3$. Additionally, in yeast, the fraction of q of nuclearly-localized to cytoplasmically localized proteins is about 1/2 (Kumar et al., 2002). However, since this factor is a major determinant of the NC volume ratio, and we find ratios that are about 0.2, we conclude that this factor must be smaller in mammalian cells.

The apparent tension of the nucleus, estimating σ_N or σ_0 is found to be around 10^{-6} N/m in nuclear shape fluctuations experiments (Chu et al., 2017; Introini et al., 2023). However, this value is an underestimation of the actual tension, and it is affected by excess flickering due to transient deformations of active origin, since fluctuating forces due to activity decrease the effective tension with respect to the actual mechanical tension (see ref. (Introini et al., 2023) for a detailed explanation). We can assume that this value is a lower bound for the real mechanical tension σ_0. As an upper bound we take the value $\sigma_0 \simeq 10^{-2}$ N/m, similar to the value 0.023 N/m found by a fit of a poroelastic model to data from pig Chondrocites (Finan et al., 2008).

We assume that cytoskeletal forces are compressive, in line with the recent literature (but we have also considered the opposite case, see below). In order to estimate an upper bound for the external tension generated by these forces, as well as an order of magnitude for cytoskeletal forces, we have considered force traction measurements for cells on substrates of different stiffness. For a substrate stiffness of 5 kPa, we measured a total traction energy of 0.5 fJ from the same cells (Nastaly et al., 2020). With this value, if we assume that all the energy is transmitted on the nucleus (i.e., an upper bound for the tension), that this energy is $\sigma_N S$, where S is the nuclear surface area. For a nucleus of radius 4 μm this gives us $\sigma_N \simeq 10^{-5}$ N/m. Vianay and coworkers (Vianay et al., 2018) find higher values (about 25 fJ) for RPE1 cells on 40 kPa substrates. This gives $\sigma_N \simeq 5 \cdot 10^{-4}$ N/m.

We can also consider the total force on a nucleus of radius 4 μm ($4 \cdot 10^{-6}$m), to exert mechanically a pressure of 1000 Pa (this pressure would be able to perturb the osmotic pressure from the nucleus, which as discussed above is of order 4 kPa). Since $\Delta P_N = 2\sigma_N/R_N$ this upper bound would correspond to an (effective) surface tension of about 10^{-3} N/m. This value is considerably higher than the values we estimate from force traction measurements, which can already be considered upper bounds. Since total force is pressure times surface, we would need about 50 nN ($50 \cdot 10^{-9}$N) from the cytoseleton to obtain this force on the whole nuclear surface (still assuming a nucleus of radius 4 μm). This is equivalent to order 50.000 motors exerting pN forces. However, these forces on the nucleus would be observed also by traction force microscopy, by Newton's third law.

Given these considerations, we have considered a range of 10^{-6} to 10^{-2} N/m for both constitutive and applied mechanical tensions, and we speculate that a realistic range for tensions on the nucleus applied by the cytoskeleton could be $10^{-5} - 10^{-4}$ N/m.

In order to fix the bias parameter in the model variant with force-biased transport, we used the data provided by Andreu and coworkers (Andreu et al., 2021). In this study (Fig 2I of the paper), the authors find a change in the concentration of nuclearly localized proteins c_N by a factor of 3/2 by using gels of stiffness from 1.5 to 30 kPa. Based on the above considerations on force traction measurements, we assume that these conditions could correspond to $\sigma^{(1)}_{\rm ext} \approx 10^{-5}$ N/m and $\sigma^{(2)}_{\rm ext} \approx 10^{-4}$ N/m respectively. Assuming as above that

$$c_N = c_N^{(0)} e^{\eta \sigma_{\rm ext}} \ , \tag{4}$$

we can now use these two conditions to calibrate this dependency, and find η. Since

$$\frac{c_N^{(2)}}{c_N^{(1)}} = \exp\left[\eta(\sigma^{(2)}_{\rm ext} - \sigma^{(1)}_{\rm ext})\right] \ , \tag{5}$$

we get $\eta \approx 4 \cdot 10^3$ m/N.

The bag-in-a-bag model may capture the behavior of hyperosmotic shocks, but cannot describe the experimental observations for cell spreading/detachment.

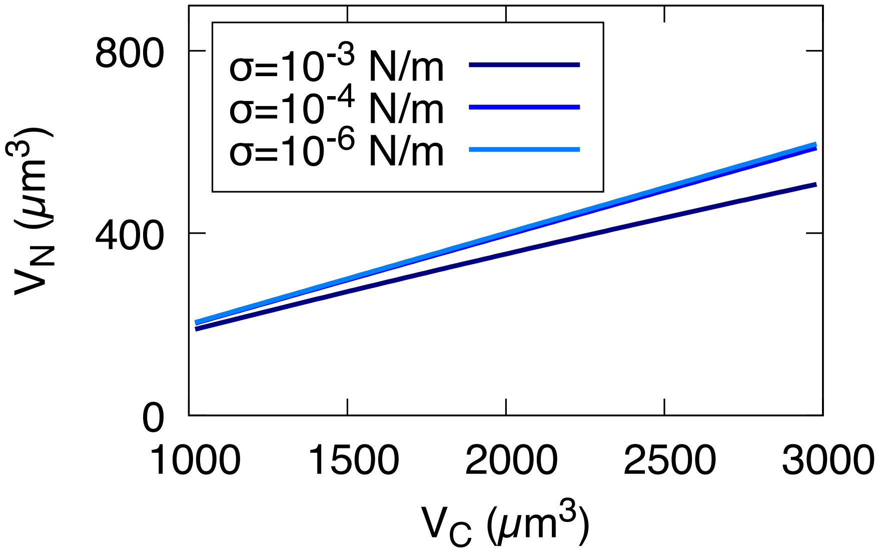

FIG. SM1: Upon osmotic shocks (on time scales of tens of minutes, before mechano-osmotic regulation takes place (Venkova et al., 2022)), the bag-in-a-bag model predicts nuclear volume to be proportional to cytoplasmic volume, and the proportionality becomes nonlinear only for strong nuclear surface tension. Parameter values: $q = 0.2$ (fraction NLS/NES proteins), σ_N in the range 10^{-6} to 10^{-3} N/m (different solid curves), $N_N = 3 \cdot 10^8$ (nuclearly localized macromolecules).

In order to capture the changes of nuclear volume during a hyperosmotic shock, we assume that extracellular ion concentration $c_{\rm out}^{\rm ions}$ changes, setting a new value for $\Pi_C = \alpha N_C/(\beta V_C)$, with $\beta = (k_B T)^{-1}$. We then solve Eq. (2) for V_N using Π_C as an input.

$$\frac{N_N}{V_N} - \alpha\beta\Pi_C = \frac{2\beta\sigma_N}{R_N} \ , \tag{6}$$

where $4/3\pi R_N^3 = V_N$.

For negligible σ_N, the solution of this equation is simply

$$V_N = \frac{N_N}{\alpha N_C} V_C := q V_C \ , \tag{7}$$

where q is the ratio between nuclearly localized ("NLS") and nuclearly exported ("NES") proteins, estimated above (from yeast data) to be close to 1/2.

For sufficiently small values of the tension [1], we can solve perturbatively the equation and

[1] Specifically, the non-dimensional quantity $2\beta\sigma_N V_N^{2/3}/N_N$ should be small, and in the range of empirically relevant parameter values that we consider this is always the case

obtain a tension-dependent expression,

$$V_N = qV_C - \frac{z}{N_N}(qV_C)^{5/3}, \tag{8}$$

where we have defined $z = 2\beta\sigma_N$.

Figure SM1 reports these expressions, for parameter values within the ranges estimated above. The plot shows that (in line with the data) the qualitative model expectation is a proportionality between nuclear volume and cytoplasmic volume, basically set by the ratio of osmotically active particles. Tension, in the range of estimated realistic values, does not affect this trend. It reduces the nuclear volume at fixed cytoplasimc volume, but the changes are only slight. Compressive forces by the cytoskeleton can contribute to this trend, but again the expected volume reduction is small. We estimate it to be at most less than 10% compared to a tensionless nucleus for the upper bounds of the realistic values for the constitutive tension and the cytoskeletal compressive forces. As a consequence, we find that the nuclear size changes predicted by such a "bag-in-a-bag" model (Deviri and Safran, 2022; Lemière et al., 2022) in presence of changes in external forces are at odds with our experimental observations in cell spreading and detachment experiments. During cell spreading, the cytoskeleton is believed to apply compressive forces to the nucleus, which, would lead to volume-decreasing deformations, in contrast with the observed increase in volume. Viceversa for cell detachment these forces should be released.

To sum up, assuming a bag-in-a-bag framework (i) the volume changes observed in osmotic shocks are explained but (ii) the assumption that tension is too small to modify volume in a considerable way at odds with the data. Crucially, in this prediction the external tension makes a *qualitative* difference. Without an applied external tension, the model is insufficient to describe the data, as it cannot explain why nuclear volume changes during cell spreading, while cytoplasmic volume is roughly constant. However, if the cytoskeleton exterts compressive forces, the model remains at odds with the data, as the nuclear volume increases upon spreading and decreases upon detachment and in suspended cells.

Force-biased transport can predict the result of spreading/detachment experiments.

Conversely, we found that extending the model to the variant including tension-biased transport could produce values comparable with our experimental data (Figure 3 in the main text and Figure SM2). In the following, we neglect direct effects of surface tension and we assume that only tension-biased transport changes the nuclear volume. This choice is conservative, as well as simplifying the model technically. In this case, nuclear volume is simply computed as

$$V_N = \frac{N_N}{\alpha N_C} V_C(1 + \eta\sigma_{\text{ext}}) = qV_C(1 + \eta\sigma_{\text{ext}}) \ , \tag{9}$$

where all the parameters are specified (see above). We can ask how big η needs to be, in order to create an increase in nuclear volume of at least 10%. This is given by the condition $\eta\ \sigma_{\text{ext}} \approx 0.1$, i.e. $\eta \approx 1/(10\ \sigma_{\text{ext}})$.

Our estimate based on data from Andreu and coworkers (Andreu et al., 2021) was that $\eta \approx 4 \cdot 10^3$ m/N. With this value, a tension $\sigma_{\text{ext}} \approx 4 \cdot 10^{-4}$ N/m would be sufficient to achieve visible nuclear deformations. This range of tension is still on the upper end of what we estimate to be achievable from force traction data, but crucially the effect of nuclear deformation on force-biased transport appears to be at least ten times stronger than the direct effect.

We note that we have assumed here that the the impact of the "internal" surface tension on the tension bias is negligible. Technically, this assumption avoids the problem of computing a self-consistent value for the nuclear volume. Biologically, it is possible to speculate that (i) the biased transport saturates at some value of the external forces and/or (ii) tension biased

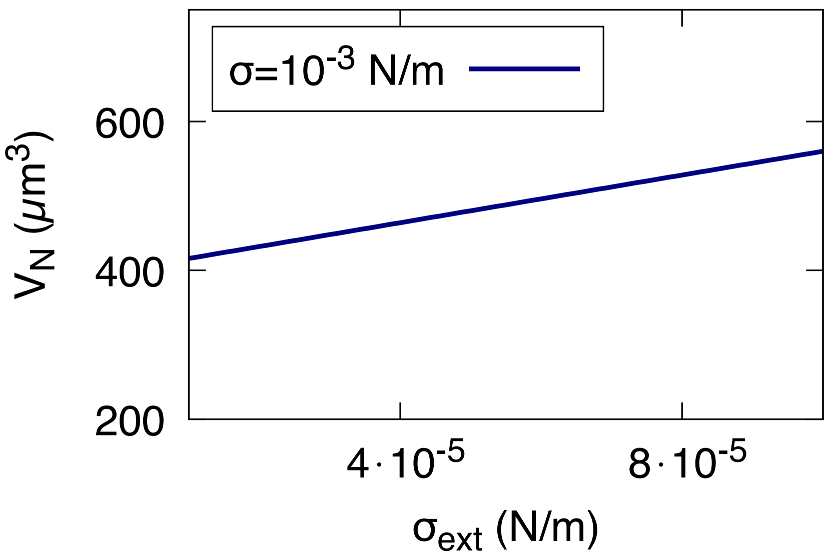

FIG. SM2: A bias on nuclear import caused by extensile tension can increase nuclear volume more strongly than direct tension, at fixed cytoplasmic osmotic pressure. The plot reports predicted nuclear volume (y axis) for increasing applied extensile tension σ_{ext}, at fixed basal nuclear surface tension σ_0. Parameter values: $q = 0.2$ (fraction NLS/NES proteins in absence of applied tension) $\sigma_0 = 0$ and direct effect of σ_{ext} on nuclear size neglected, $\eta = 4 \cdot 10^3$ m/N (tension-transport bias), $V_C = 2000$ μm^3 (cytoplasmic volume).

transport may show "adaptation", in the sense that it is zero in a resting condition (even in presence of some external forces), then responds to external forces perturbing that condition. All these ingredients could be added to the model in a straightforward way. However, given the proof-of-principle aim of our modeling effort, and in absence of precise data to elucidate all these mechanisms, we decided to leave these additional ingredients out of our description. Crucially, the key ingredient of the model is again qualitative: force-biased transport can explain why nuclear volume increases during cell spreading also under the assumption that the cytoskeletal forces felt by the nucleus upon cell spreading are compressive. The model can also explain the qualitative trend of the cytoskeletal perturbations in our experiments, as the release of cytoskeletal compressing forces would remove the force-biased transport, reducing nuclear size.

The direct effect of putative extensile forces is expected to be small.

While cytoskeletal forces are generally believed to be compressive, one can also wonder whether cell spreading could associate to extensile forces which directly deform the nucleus, and cell detachment to the release of these forces. To address this question, we also considered the case of $\sigma_{\text{ext}} < 0$, wich in our formalism can describe extensile external forces. In order to explore the effect of an applied (extensile) tension, we solved Eq. (2) taking explicitly into account the external tension σ_{ext} and once again for fixed cytoplasmic osmotic pressure Π_C.

In this case it is simple to obtain an implicit expression of $\sigma_{\text{ext}}(V_N)$ and then get the inverse function by implicit plot,

$$-\sigma_{\text{ext}} = \sigma_0 + \frac{1}{2}\left(\Pi_C V_N^{1/3} - N_N V_N^{-2/3} k_B T\right) \tag{10}$$

Figure SM3 shows that, according to the bag-in-a-bag model, only (very strong) externally applied forces giving rise to extensile tension of order $10^{-3} - 10^{-2}$ N/m can noticeably affect

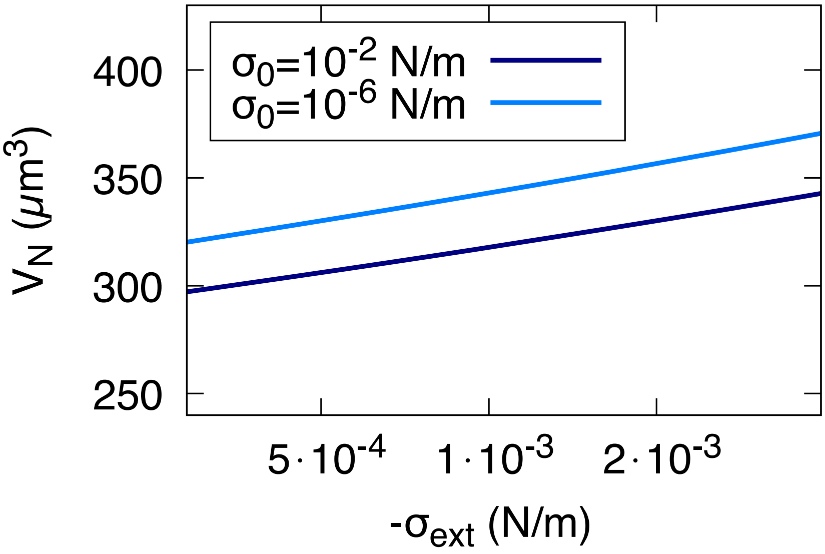

FIG. SM3: An externally applied extensile tension can mildy increase nuclear volume, at fixed cytoplasmic osmotic pressure. The plot reports predicted nuclear volume (y axis) for increasing applied extensile tension $\sigma_{\rm ext}$, at fixed basal nuclear surface tension σ_0. Parameter values: $\alpha = 2/3$ (fraction of osmotically active proteins that are in the cytoplasm), σ_0 in the range 10^{-6} to 10^{-2} N/m (different solid curves), $\Pi_C = 4$ kPa (cytoplasmic osmotic pressure).

nuclear size (in the whole range of possibly relevant constitutive nuclear tensions).

Hence, in spreading experiments (and within the assumptions of our modeling framework), only the presence of an unrealistic increasing extensile force would explain the observed increase of nuclear volume. Consequently, we conclude that adding a strong extensile tension to the bag-in-a-bag framework leads to the prediction of enormous values of the tension to explain our data, which appears unrealistic.

References

Ion Andreu, Ignasi Granero-Moya, Nimesh R. Chahare, Kessem Clein, Marc Molina Jordàn, Amy E. M. Beedle, Alberto Elosegui-Artola, Xavier Trepat, Barak Raveh, and Pere Roca-Cusachs. Mechanosensitivity of nucleocytoplasmic transport. *BioRxiv*, July 2021. doi: 10.1101/2021.07.23.453478. URL https://doi.org/10.1101/2021.07.23.453478.

Fang-Yi Chu, Shannon C. Haley, and Alexandra Zidovska. On the origin of shape fluctuations of the cell nucleus. *Proceedings of the National Academy of Sciences*, 114(39):10338–10343, September 2017. doi: 10.1073/pnas.1702226114. URL https://doi.org/10.1073/pnas.1702226114.

Dan Deviri and Samuel A. Safran. Balance of osmotic pressures determines the nuclear-to-cytoplasmic volume ratio of the cell. *Proceedings of the National Academy of Sciences*, 119(21), May 2022. doi: 10.1073/pnas.2118301119. URL https://doi.org/10.1073/pnas.2118301119.

John D. Finan, Kevin J. Chalut, Adam Wax, and Farshid Guilak. Nonlinear osmotic properties of the cell nucleus. *Annals of Biomedical Engineering*, 37(3):477–491, December 2008. doi: 10.1007/s10439-008-9618-5. URL https://doi.org/10.1007/s10439-008-9618-5.

Viola Introini, Gururaj Rao Kidiyoor, Giancarlo Porcella, Pietro Cicuta, and Marco Cosentino Lagomarsino. Centripetal nuclear shape fluctuations associate with chromatin condensation in early prophase. *Communications Biology*, 6(1), July 2023. doi: 10.1038/s42003-023-05074-9. URL https://doi.org/10.1038/s42003-023-05074-9.

Dong-Hwee Kim, Bo Li, Fangwei Si, Jude Philips, Denis Wirtz, and Sean X. Sun. Volume regulation and shape bifurcation in the cell nucleus. *Journal of Cell Science*, January 2015. doi: 10.1242/jcs.166330. URL https://doi.org/10.1242/jcs.166330.

Anuj Kumar, Seema Agarwal, John A. Heyman, Sandra Matson, Matthew Heidtman, Stacy Piccirillo, Lara Umansky, Amar Drawid, Ronald Jansen, Yang Liu, Kei-Hoi Cheung, Perry Miller, Mark Gerstein, G. Shirleen Roeder, and Michael Snyder. Subcellular localization of the yeast proteome. *Genes & Development*, 16(6):707–719, March 2002. doi: 10.1101/gad.970902. URL https://doi.org/10.1101/gad.970902.

Jol Lemière, Paula Real-Calderon, Liam J Holt, Thomas G Fai, and Fred Chang. Control of nuclear size by osmotic forces in schizosaccharomyces pombe. *eLife*, 11, July 2022. doi: 10.7554/elife.76075. URL https://doi.org/10.7554/elife.76075.

Ron Milo. What is the total number of protein molecules per cell volume? a call to rethink some published values. *BioEssays*, 35(12):1050–1055, 2013. doi: https://doi.org/10.1002/bies.201300066. URL https://onlinelibrary.wiley.com/doi/abs/10.1002/bies.201300066.

T. J. Mitchison. Colloid osmotic parameterization and measurement of subcellular crowding. *Molecular Biology of the Cell*, 30(2):173–180, January 2019. doi: 10.1091/mbc.e18-09-0549. URL https://doi.org/10.1091/mbc.e18-09-0549.

Paulina Nastaly, Divya Purushothaman, Stefano Marchesi, Alessandro Poli, Tobias Lendenmann, Gururaj Rao Kidiyoor, Galina V. Beznoussenko, Stefania Lavore, Orso Maria Romano, Dimos Poulikakos, Marco Cosentino Lagomarsino, Alexander A. Mironov, Aldo Ferrari, and Paolo Maiuri. Role of the nuclear membrane protein emerin in front-rear polarity of the nucleus. *Nature Communications*, 11(1), May 2020. doi: 10.1038/s41467-020-15910-9. URL https://doi.org/10.1038/s41467-020-15910-9.

Romain Rollin, Jean-François Joanny, and Pierre Sens. Physical basis of the cell size scaling laws. *eLife*, 12, May 2023. doi: 10.7554/elife.82490. URL https://doi.org/10.7554/elife.82490.

G Salbreux, J F Joanny, J Prost, and P Pullarkat. Shape oscillations of non-adhering fibroblast cells. *Physical Biology*, 4(4):268–284, November 2007. doi: 10.1088/1478-3975/4/4/004. URL https://doi.org/10.1088/1478-3975/4/4/004.

Larisa Venkova, Amit Singh Vishen, Sergio Lembo, Nishit Srivastava, Baptiste Duchamp, Artur Ruppel, Alice Williart, Stéphane Vassilopoulos, Alexandre Deslys, Juan Manuel Garcia Arcos, Alba Diz-Muñoz, Martial Balland, Jean-François Joanny, Damien Cuvelier, Pierre Sens, and Matthieu Piel. A mechano-osmotic feedback couples cell volume to the rate of cell deformation. *eLife*, 11, April 2022. doi: 10.7554/elife.72381. URL https://www.biorxiv.org/content/early/2021/07/21/2021.06.08.447538.

Benoit Vianay, Fabrice Senger, Simon Alamos, Maya Anjur-Dietrich, Elizabeth Bearce, Bevan Cheeseman, Lisa Lee, and Manuel Théry. Variation in traction forces during cell cycle progression. *Biology of the Cell*, 110(4):91–96, March 2018. doi: 10.1111/boc.201800006. URL https://doi.org/10.1111/boc.201800006.
