# Supplementary material for: N2FXm, a method for joint nuclear and cytoplasmic volume measurements, unravels the osmo-mechanical regulation of nuclear volume in mammalian cells
Source: Nat Commun. 2024 Feb 7;15:1070. doi: 10.1038/s41467-024-45168-4 (PMC10850064; doi:10.1038/s41467-024-45168-4)
Supplement: Supplementary file 3 — Description of additional supplementary files [file 41467_2024_45168_MOESM3_ESM.pdf]

DESCRIPTION OF ADDITIONAL SUPPLEMNTARY FILES DOCUMENT

Supplementary Movie 1: Example of cell and nuclear volume curves generation for a RPE1 cell dividing twice during the acquisition. Here, the 3 fluorescent channels are merged (BFP, GFP and Texas Red) and all the ROIs defining the cell area reported.
